# REST framework: A modelling approach towards cooling energy stress mitigation plans for future cities in warming Global South

**DOI:** 10.1016/j.scs.2020.102315

**Published:** 2020-10

**Authors:** Ronita Bardhan, Ramit Debnath, Joao Gama, Upadhi Vijay

**Affiliations:** a Behaviour and Building Performance Group, Department of Architecture, University of Cambridge, CB2 1PX, United Kingdom; b Centre for Urban Science and Engineering, Indian Institute of Technology Bombay, Mumbai, 400076, India; c Energy Policy Research Group, Judge Business School, University of Cambridge, CB2 1AG, United Kingdom; d Laboratory of Artificial Intelligence and Decision Support, and Faculty of Economics, University of Porto, Porto, 4099 002, Portugal; e Civil and Environmental Engineering Department, University of California Berkeley, Berkeley, CA, 94720, USA

**Keywords:** Smart cities, Energy demand, Urban heat island, Cooling policy, Distributive justice, India

## Abstract

Future cities of the Global South will not only rapidly urbanise but will also get warmer from climate change and urbanisation induced effects. It will trigger a multi-fold increase in cooling demand, especially at a residential level, mitigation to which remains a policy and research gap. This study forwards a novel residential energy stress mitigation framework called REST to estimate warming climate-induced energy stress in residential buildings using a GIS-driven urban heat island and energy modelling approach. REST further estimates rooftop solar potential to enable solar photo-voltaic (PV) based decentralised energy solutions and establish an optimised routine for peer-to-peer energy sharing at a neighbourhood scale. The optimised network is classified through a decision tree algorithm to derive sustainability rules for mitigating energy stress at an urban planning scale. These sustainability rules established distributive energy justice variables in urban planning context. The REST framework is applied as a proof-of-concept on a future smart city of India, named Amaravati. Results show that cooling energy stress can be reduced by 80 % in the study area through sensitive use of planning variables like Floor Space Index (FSI) and built-up density. It has crucial policy implications towards the design and implementation of a national level cooling action plans in the future cities of the Global South to meet the UN-SDG – 7 (clean and affordable energy) and SDG – 11 (sustainable cities and communities) targets.

## Highlights

• Higher cooling energy stress due to future urbanisation in Global South.
• Urban energy stress modelling using an integrated geo-spatial energy network.
• Residential decentralised energy system as grid energy stress management strategy.
• Distributive energy justice through urban planning and peer-to-peer energy sharing.

## 1 Introduction

Climate change is warming cities of Global South that poses severe health and well-being risks if the cooling needs are not met. Warmer cities demand more energy-intensive solutions to cool their citizens, the accurate estimation of which remains uncertain in the current literature. Defining a good ‘cooling’ energy policy for the Global South remains a critical challenge across the vertices of governance, policymaking, research and industry to mitigate climate-change-induced effects (Ozawa, Chaplin, Pollitt, Reiner, & Warde, 2019; TheWorldBank, 2018). This study forwards an energy stress mitigation framework (called, REST) to effectively allocate energy for increasing cooling needs at a residential neighbourhood-level using rooftop solar photovoltaics. The REST framework is envisaged within the scope of improving the quality of life in middle and low-income residential apartments, as per the dialogue of eudemonic well-being effects of distributive energy justice (Samarakoon, 2019; National Institute of Urban Affairs, 2016). In this purview, we define energy stress as ‘the shortage of electricity to meet urban-residential cooling needs under the warming effects of climate change’. The REST framework is intended to aid policymakers, planners and researchers in allocating appropriate renewable resources to meet the future cooling needs. It has specific significance in the policy design process of national cooling action plans. Here, we draw critical policy implications for the national cooling plan of India (Ministry of Environment Forest & Climate Change, 2019).

The national cooling action plan of India (known as India Cooling Action Plan (ICAP)) provides a 20-year perspective (2017−18 to 2037−38) and recommendations. It forwards a policy roadmap to address the sustainable cooling requirements across sectors in India (Ministry of Environment Forest & Climate Change, 2019). The ICAP categorises space cooling in buildings as a critical segment of the cooling demand in the country, and stresses on the importance of climate resilience building design for addressing future cooling needs. ICAP specifically highlights the importance of sustainable urban planning in addressing future cooling demand under national missions like Housing for All (Ministry of Housing and Urban Affairs, 2020a; Rathore et al., 2013), Smart Cities (Ministry of Housing and Urban Affairs, 2020a) and Solar Cities (Kandt, 2012). These missions cater to the rising middle-class in India who are the major force behind rapid urbanisation. The proposed REST framework is designed to aid planners and decision-makers in deriving ‘good’ cooling energy policies for future smart cities under climate-change and urbanisation-induced heat stresses.

In a similar energy modelling effort, Mastrucci, Byers, Pachauri, and Rao (2019) have predicted the future residential cooling demand on a global grid and concluded that interventions are required at neighbourhood and city-level through urban planning to meet the cooling targets under a warming climate. This study forwards their analysis by investigating cooling potential and energy stress at a neighbourhood level in a *to-be* smart city in a hot and humid region in India. The novelty of this study lies in the methodological innovation towards identifying cooling energy stress at a neighbourhood level using GIS-based urban heat island (UHI) simulation. This novelty is extended by the cooling policy modelling approach that uses decentralised renewable solutions (rooftop solar photovoltaics (PV)) in a peer-to-peer residential energy network. The strength of the proposed framework lies in its replicability for cooling action-planning across rapidly urbanising and warming cities, where majority of the building stocks are yet to be built.

Besides, the methodological approach towards identification and mitigation of residential cooling energy stress due to UHI-effects creates an evidence-based policy modelling approach for distributive energy justice. Lack of such approach for distributive justice planning remains a literature gap (Debnath, Darby, Bardhan, Mohaddes, & Sunikka-Blank, 2020). Besides, the context of *to-be* cities adopted here refers to future smart cities which already have a defined master plan and are in the construction phase under the Smart Cities Mission of the Government of India (Ministry of Housing and Urban Affairs, 2020a)(). Our examination adds value to the cooling energy policy making process and provides a proof-of-concept generation platform for testing various short-term, moderate-term and long-term policy objectives of the ICAP for smart cities and residential energy estimations (Ministry of Environment Forest & Climate Change, 2019).

In doing so, we investigate the influence of sensitive planning variables like Floor Space Index (FSI) and built-up area on cooling energy stress due to UHI-effects on a *to-be built* smart city of India. As a distributive energy justice solution, we use renewable solutions like rooftop solar-PV as per ICAP’s recommendation. And a decentralised peer-to-peer energy sharing optimisation scheme to derive key sustainable urban planning rules. Therefore, the primary research question of this study is, ‘how do we allocate renewables to meet future cooling demand in the residential neighbourhood due to UHI and climate change effect?’. The following objectives were formed to address the primary question,

• To simulate urban heat island effect in a *to-be*-built smart city from Landsat 8 data and building footprint modelling.
• To estimate residential cooling energy demand due to UHI-induced warming effects in a low and middle-income neighbourhood.
• To determine rooftop solar potential to mitigate energy stress due to cooling needs, as a distributive energy justice solution
• To derive an optimised decentralised residential solar PV network and a sustainability planning guideline for future warming cities.

This study is organised in the following sections, Section 2 provides a detailed background on neighbourhood scale energy modelling framework and assumptions posed in the India Cooling Action Plan for the building sector. The subsection 2.1 presents a systematic literature review urban heat island and residential energy demand modelling approaches under the uncertainties of climate change. The Section 2.2 illustrates current policy modelling efforts in national cooling action plans and the influence of distributive energy justice in it. Section 2.3 further explains the India Cooling Action Plan for the building sector in detail and present a case study of the *to-be built* smart city in India. The detailed methodological framework of the proposed of the proposed REST framework is illustrated in Section 3. The Results and Discussion on energy stress and sustainability rules for mitigation of the energy stress is illustrated in Section 4. The conclusion, future work and limitation of the study is in Section 5.

## 2 Background

### 2.1 Urban heat island, climate change and residential energy demand

Urban heat island (UHI) refers to the development of higher urban temperature as compared with surrounding rural areas (Oke, 1981). The literature on UHI estimation shows that it is a combined effect of urban morphology, thermal admittance, anthropogenic heat and sky radiation (Montaveza, Gonzalez Roucob, & Valerob, 2007). UHI is fundamentally a manifestation of land surface temperature (LST) and is often an indication of rates of urbanisation (Parvez, Aina, & Balogun, 2019; Rasul et al., 2017) LST and UHI are linked through various parameters like land cover (impervious vs pervious surfaces), urban morphological features and surface properties. While previous researches have shown that that higher LSTs correspond to higher urban intensity or imperviousness, the lower values are generally related to higher vegetation cover (Weng, 2012). Thus, indicating that with increased urbanisation i.e. conversion of agricultural lands to urban uses can implicitly increase LST. However, some studies on the UHI and LST in various climatic zones indicate that in dry arid regions with high LST might produce cool islands through the inversion of UHI phenomenon (Lazzarini, Marpu, & Ghedira, 2013). Yet, there is a general consensus that in tropical climate zones higher LST is directly correlated to intensified UHI (Wang et al., 2019). Several researches have verified that there are synergistic interactions between UHI and climate change related heating and can potentially exacerbate the heating effects of climate change, thus leading to ‘heating-up’ cities rapidly (He et al., 2020; Iping, Kidston-Lattari, Simpson-Young, Duncan, & McManus, 2018). In India a study of 44 cities having more than one million population, showed that the night-time urban heat island intensity was positive throughout the year. This suggested slow warming of urban and rural areas indicating the higher impact of global warming (Raj, Paul, Chakraborty, & Kuttippurath, 2019).

The rise in ambient temperature has a significant impact on the electricity consumption of the residential sector, increasing the peak and total electricity demand substantially (Santamouris, Cartalis, Synnefa, & Kolokotsa, 2015). Occupants will purchase more energy-intensive cooling devices to mitigate the rise in temperature that will put tremendous pressure on already strained electricity grids. While this may reflect the realities of developing nations, the distribution of energy resources in the rapidly urbanising context of Global South remains inequitable (Wang & Kintrea, 2019). The allocation of future cooling resources in warming global south must address the inequality in energy access, availability and affordability and create resilient pathways to foster distributive energy justice (Guruswamy, 2015).

The relations between daily energy consumption and the correspondent ambient temperature is not linear and presents high seasonality variation. The curve of electricity demand obtains its peak value during the warmest summer period (Santamouris et al., 2015). For India, the temperature elasticity of the electricity demand was found to be close to 1.7 % in 2007 (De Cian, Lanzi, & Roson, 2007). Since then the pace of urbanisation and industrialisation has changed dramatically with more low- and middle-income communities migrating to cities, that have aggravated the UHI driven heat waves on Indian cities (Sharma, Hooyberghs, Lauwaet, & De Ridder, 2019). Heatwave mitigation strategies include modifying the urban micro-climate through built environment changes like geometric aspect, cool roof, urban green spaces and land-use change (Salata et al., 2017). However, these strategies are yet to receive applied attention in the policy sphere in India. The most common response to the rise in ambient temperature is the purchase of energy-intensive cooling devices like air conditioners (AC). So much so that space cooling devices (AC, air-coolers and fans) have become the second-highest energy consuming segment by end-use in India (Chunekar, Varshney, & Dixit, 2016). It is predicted that the residential energy demand in India will increase by more than eight times by 2050 with increment in aggregated floor area by five times (Shukla et al., 2015). These studies do not explicitly evaluate the embedded UHI and climate change effects on the rise in the cooling energy demand, and it remains a literature gap.

The Residential Energy Stress Mitigation (REST) framework proposed in this study mainly contributes to this literature gap by empirically investigating UHI-driven heat stress and its effect on cooling demand using an analytical UHI model of (Montaveza et al., 2007). A neighbourhood-scale cooling energy demand estimation using a cooling-degree day (CDD)-based analytical model adapted from (Fichera, Inturri, La Greca, & Palermo, 2016). The context of the *to-be*-built city presented in this study accentuates the need for demand-side energy sustainability consideration in the city planning agenda of Global South. Existing literature presents a wide array of urban-scale building energy modelling approaches that include GIS-based, analytical and computational-simulation based approaches (Reinhart & Cerezo Davila, 2016). The REST framework explains the need of future cities to be modelled with the expected outcomes of climate change.

Mastrucci et al. (2019) have predicted the future residential cooling demand on a global grid under climate change warming effects using an analytical variable degree-day (VDD) method and made a comparison with energy modelling engine, EnergyPlus (EPLUS). They mention that urban planning and energy allocations must work in tandem right from individual and neighbourhood-level to establish distributive energy justice through renewable energy services. Kaden and Kolbe (2013) and Strzalka et al. (2011) have used semantically enriched 3D city models and suggested the use of an energy balance equation for a better estimate of city-scale heating energy demand. We adopt a similar approach to estimate neighbourhood level cooling demand for the *to-be*-built city that lacks historical information concerning urban morphology and energy use.

### 2.2 Decentralised energy planning as distributive energy justice in heat stress

Distributive energy justice is concerned with the outcomes of decision making (i.e. cost and benefits) and their welfare distribution (Jenkins, McCauley, & Forman, 2017; McCauley et al., 2018; Mundaca, Busch, & Schwer, 2018; Sovacool & Dworkin, 2014). Recent developments on practical approaches have used the spatial characteristics of the distributive justice concepts (Bouzarovski & Simcock, 2016; Lacey-Barnacle, Robison, & Foulds, 2020). Poruschi and Ambrey (2018) have used spatial planning-based approach in understanding the impact of built environment and feed-in tariffs on the installation of rooftop solar photovoltaic (PV) in Australia’s capital cities. This study examined the distributional benefits of solar PV on heating and cooling energy demand across the urban built environment with varying density. The conclusions indicated on the importance of decentralised planning to meet future heating and cooling demands in highly dense urban areas. Similarly, Grover and Daniels (2017) spatially explored the distributional injustices associated with the feed-in tariff program for clean energy deployment in England and Wales. They focussed on understanding the distributional impacts of non-income drivers like social class, dwelling type, home ownership status, settlement density and local information spillovers of clean energy technologies (Grover & Daniels, 2017). The proposed REST framework builds on such contemporary spatial approaches to distributive justice using decentralised renewable solutions

Mastrucci et al. (2019) found that up to 4.1 billion people need access to indoor cooling technologies to avoid heat related stress, especially in India, South East Asia and Sub-Saharan Africa. The occurrence of heat waves are likely magnified in urban areas because of heat island effects which has a severe health and well-being burden (Campbell-Lendrum & Corvalán, 2007). The middle and low-income households are especially susceptible to heat-stress related health impacts that can be mitigated through equitable cooling policies (Mastrucci et al. (2019, Mazzone, 2020). Decentralised renewable systems (also known as distributed energy resource (DER) systems) have garnered a lot of interest as a tool to reduce such equity gap related to energy accessibility and affordability (Chmutina, Wiersma, Goodier, & Devine-Wright, 2014; Heffron, McCauley, & Sovacool, 2015; Johnson & Hall, 2014; Tomei & Gent, 2015). The DERs are typically located at the consumer site and offers the potential of integration and management of dispatchable and non-dispatchable energy sources to satisfy consumer demand. It has the advantage of addressing the intermittent and predictable nature of renewable energy (Luo, Liu, Liu, & Liu, 2019).

Literature illustrates a wide-array of optimisation methods for DERs, Malliotakis and Founti (2017) utilised a custom-built simulation tool (DEPOSIT) to assess the energetic performance of district cooling and heating units in a micro-Combined heat and power generation DER context. The core-engine of DEPOSIT was based on TRYNSYS model. A series of in-depth investigation on DER was conducted by Sameti and Haghighat (2019a, 2019b) and Sameti and Haghighat (2018) using mixed integer linear programming (MILP) model to simultaneously optimise the layout, capacity and operation of district heating and cooling network. More recently, Luo et al. (2019) have used a game-theoretic approach to optimise design and cost allocation of a DER with district energy network for an isolated island in South China Sea. While these models and approaches assume a larger spatial scope (i.e. district level), we limit our scope to a single residential neighbourhood of a future smart city that already has mandated rooftop solar PV as the primary decentralised energy system (see Section 2.3). In doing so, we focus heavily on mitigating energy stress due to increase cooling demand due to urban heat island formation. To enable distributive energy justice in this energy stress mitigation process, we employ a custom peer-to-peer sharing algorithm of excess renewable energy (solar PV) within the neighbourhood (see Section 3.6 for more details). For a comprehensive review on the state-of-the-art methods in peer-to-peer DER sharing, see Sousa et al. (2019). More recently, Luo et al. (2019) have used a game-theoretic approach to optimise design and cost allocation of a DER with district energy network However, these models catered to buildings at a neighbourhood scale that had the need for both heating and cooling energy needs, and the calculations were performed on a theoretical scale. For our study, we lay our assumptions on the objectives of Smart Cities Mission (Ministry of Housing and Urban Affairs, 2020a)() and Solar Cities Mission (Kandt, 2012) as per the Indian Cooling Action Plan

### 2.3 India cooling action plan and residential space cooling: conventions for the REST framework

**Fig. 1:**
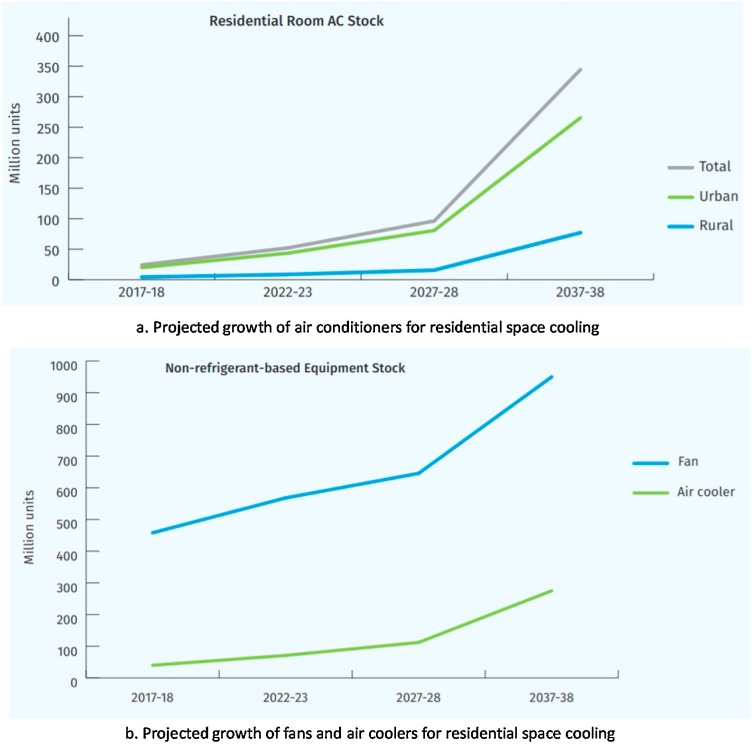
Projected space cooling demand in India for 2017 - 2038, as per the India Cooling Action Plan, Ministry of Environment, Forestry & Climate Change, Government of India (source: Ministry of Environment Forest & Climate Change, 2019).

India Cooling Action Plan (IACP) is a policy roadmap that provides a 20-year perspective (2017−18 to 2037−38) and recommendation on sustainable cooling across sectors. It is prepared by the Ministry of Environment, Forest and Climate Change, Government of India to address future cooling needs concerning climate change mitigation and sustainable development goals of the nation (Ministry of Environment Forest & Climate Change, 2019). In this study, we specifically focus on residential space cooling needs. The ICAP estimated that in 2017, the penetration of air conditioners in India households was approximately 8%. It is anticipated to rise to 21 % in 2027−28 and 40 % in 2037−38 (see Fig. 1a), with a significant ownership variability among the rural and urban households (Ministry of Environment Forest & Climate Change, 2019). Fans and air-coolers also form a significant portion of space cooling devices in India households and its share is projected rise as per the climate scenarios for 2027−28 and 2037−38 (see Fig. 1b) (Ministry of Environment Forest & Climate Change, 2019). It indicates that the space cooling demand through fans and air coolers will remain significant even in long-term scenario with added energy intensity from air conditioners.

To address the space cooling demands, the ICAP strongly recommends strict compliance to National Building Code, Model Building Bye-Laws and green building rating systems (Ministry of Environment Forest & Climate Change, 2019), pp 21. Policy recommendations include emphasis on building sustainability through climate responsive architecture that are low-cost and energy efficient. Bureau of Energy Efficiency (BEE), an agency of the Government of India that develop programs for energy conservation and efficiency in India, is preparing the Energy Conservation Building Code – Residential (ECBC – R) Part 1. The ECBC-R will forward design guidelines for energy efficient building envelops that can aid in reducing heat transfer through envelope. ICAP vetoed it by stating that ‘this intervention can help in reducing the sharply rising cooling demand and delay the purchase of first room air conditioner’ (Ministry of Environment Forest & Climate Change, 2019, pp 21).

The ECBC-R is particular critical to national programs such as under Pradhan Mantri Awas Yojana (PMAY) with the objective of affordable housing for all (Ministry of Housing & Urban Affairs, 2020a). It aims to house economically weaker section (EWS) and low-income group (LIG) population. Sustainable building guidelines (ECBC-R) can provide thermal comfort for all, reduce cooling load, and provide gains in terms of energy efficiency. In addition, the ICAP provides the following short-term recommendations for future-proofing building stocks (Ministry of Environment Forest & Climate Change, 2019),

• Promote wider penetration of climate response buildings through passive cooling and building envelop modifications to reduce cooling loads in the existing and future cities.
• Enable thermal comfort and energy saving in LIG and EWS building stocks as a distributive justice measure. Strategies should include enforcing efficient building envelope guidelines from ECBC-R, cool-roofs, decentralised renewable based energy systems and localised heat action plans.
• Minimising active cooling load through energy efficient building design as per ECBC and National Building Codes.
• Nation-wide adoption and enforcement of ECBC-R codes through the development of city-level action plans.
• Aggressive market awareness campaign on distributive justice benefits of efficient buildings.

These recommendations provide the theoretical foundation for the proposed REST framework. The REST framework further adds the effect of urban heat island (UHI) in energy stress in residential urban areas of the *to-be built* city. In doing so, our proposed framework enables proof-of-concept creation of future cities to test the efficacy of national cooling action plans, especially in areas where a significant portion of building stocks is yet to be built.

## 3 Methodology

The core methods used in this study included GIS-based estimation of land surface temperature that feeded into the urban heat island (UHI) simulation model. The temperature data from the UHI simulations were then used for the estimation of cooling energy demand in the simulated residential neighbourhoods in the study area (illustrated in Section 3.1). It was assumed that the rise in outdoor temperature due to UHI effects from urbanisation and rise in building stock would peak cooling demand in these households. And the failure to meet this demand would have a negative impact on the overall health and well-being of the middle and low-income occupants. These assumptions were in coherence with the policy recommendations of India Cooling Action Plan (ICAP), as mentioned in Section 2.3. Besides, linking the UHI-driven cooling demand with the well-being impacts further expanded the practical implications of the theories of distributive energy justice (as discussed in Section 2.2).

**Fig. 2:**
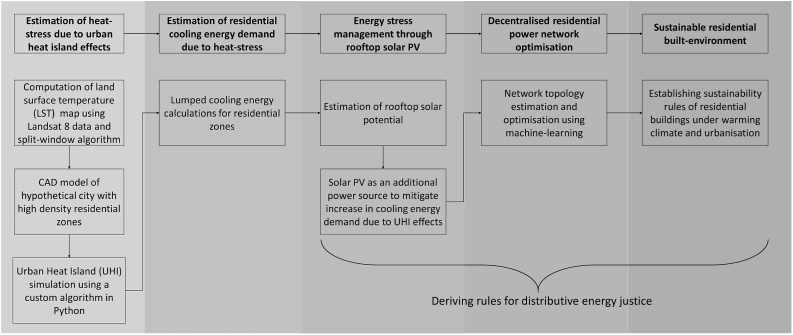
Residential Energy Stress (REST) Mitigation Framework under climate-change induced heat stress.

This study, thus, provides a framework to estimate and mitigate the energy stress from warming cities due to pressures of urbanisation and climate change. As mentioned in Section 1, our proposed framework fills the literature gap on the lack of evidence-based policy modelling framework for managing space cooling demand in low and middle-income residential houses of the Global South. The mitigation strategy was envisioned around rooftop solar photovoltaics (PV) and optimisation of a peer-to-peer decentralised renewable power network. Provisioning of such solar PV based decentralised energy solutions is believed to provide the much-needed distributive energy justice in warming future cities (a detailed literature review is presented in Section 2.3). The residential energy stress mitigation framework (REST) proposed in this study is illustrated in Fig. 2 in as a seven-stage process. The full methodological details are presented through Sections 3.1–3.7.

### 3.1 Generation of land surface temperature (LST) map

The *to-be built* smart city under study is called Amaravati. It is in the hot and humid Deccan region of India, which is prone to multiple heatwaves. The Land Surface Temperature (LST) mapping of the area where the future smart city would be located, could help in the identification of heat stress (Bick, Bardhan, & Beaubois, 2018). The LST of the study area was retrieved using the Landsat 8 TIRS data of March 15, 2019, containing the TIR bands (Band 10 and 11) and OLI bands (Band 2,3, 4 and 5). March was chosen as it represented a typical summer month in the region when people would usually use space cooling devices like fans, air coolers and air conditioners (ACs). Additionally, it was the only date when a cloud-free image was available. We couldn’t chose the hottest month of May, as the satellite data for May available had high cloud cover and data gaps. To improve the robustness of our results we compared the LST with the available historical data for March 8, 2018 (see Section 4, Fig. 6 for more details).

The LST was extracted through the split-window (SW) algorithm as proposed by Rongali, Keshari, Gosain, and Khosa (2018) in ArcGIS v10.7.1. The algorithm utilised the atmospheric window in the range of 10 μm–12 μm wavelengths for the TIRS bands (10 and 11). Along with the TRIS data, the algorithm also used the OLI bands for estimating LST. Eq. (1) represents the mathematical structure for estimation of LST.

(1) $$T_{s}=T_{i}+c_{1}\left(T_{i}-T_{j}\right)+c_{2}{\left(T_{i}-T_{j}\right)}^{2}+C_{0}+\left(c_{3}+c_{4}w\right)\left(1-\varepsilon \right)+(c_{5}+c_{6}w)\text{Δ}\varepsilon$$

where $T_{s}$ is the land surface temperature, $T_{i}$ and $T_{j}$ are atmospheric-sensor TB of the SW bands i and j in Kelvin, ε is the mean emissivity, Δε is emissivity difference, w is the total atmospheric water vapor content (g/cm^2^), and $c_{0}$ to $c_{6}$ are the SW coefficients which were determined from the simulated data. Here $T_{i}$ and $T_{j}$ corresponds to the brightness temperature of bands 10 and 11 in Kelvin(K).

The OLI sensor bands 2,3,4, and 5 were stacked, and the normalized difference vegetation index (NDVI) was generated using the bands 4 and 5. The NDVI image was then used to extract the fractional vegetation cover (FVC) of the study area, which was dependent on the emissivity of the soil and vegetation for bands 10 and 11. The NDVI values calculated from the given bands for soil and vegetation were 0.012 and 0.19, respectively. These values, along with the FVC image was then used to compute the land surface emissivity (LSE) image. The LSE measured the ability of the study area to convert thermal energy or heat energy into the radiant energy. Further estimations of these values were made based on the land cover and land-use features of the study area, as presented in (Bardhan, Debnath, & Bandopadhyay, 2016; Li, Zhao, & Deng, 2015; Sahana, Ahmed, & Sajjad, 2016). The emissivity values for Indian soil was taken from (Rajeshwari & Mani, 2014).

Although the city development will contribute to the urban heat, a part of it will be mitigated by the planned landscaped areas and green spaces. However, it was difficult to estimate the effect of green cover on temperature in absence of details regarding the type and species of green cover planned in Amaravati (i.e the resultant NDVI). Previous studies have shown that apart from local climate and quality of green space, the size and geometric shape of the green cover had a bearing on the urban heat island (UHI) intensity reduction (Aram, Higueras, Solgi, & Mansournia, 2019; Chen et al., 2012). For green spaces, whose green area percentage is more than 69 % and the aspect ratio is close to 1, the cooling effect distance for large size parks (more than 10 ha (ha)) was 300 m (m) beyond the green space boundary and could reduce temperature by 1.9 °C. Whereas, if the green areas were smaller than 1 ha, such spaces had nearly negligible temperature cooling effects on their surrounding environment (Aram et al., 2019; Chen et al., 2012). Since the largest green space within our study area was 4 m^2^, we assumed that the heating mitigation capacity of such space would be negligible in alleviating mechanical space cooling demands by residents.

### 3.2 Building model for the *to-be built* smart city

**Fig. 3:**
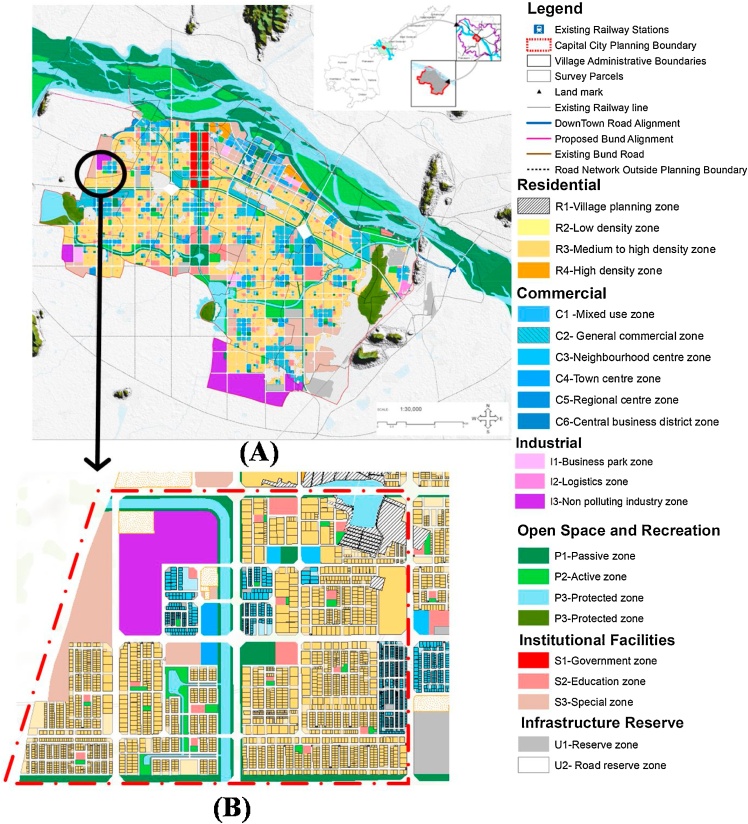
(A) Land use map of Amaravati Capital City (Source: https://gis.apcrda.org/lps/index.html) (B) Region chosen for the study (R3 Medium to high density zone).

The *to-be-*built smart city of Amaravati currently exists in its rural form. It is the proposed capital smart city of Andhra Pradesh, India that would span over 217.23 km^2^ to house a population of 4.5 million by 2050 (see Fig. 3A). Amaravati has a Köppen Climate Classification subtype "Aw", i.e., ‘Tropical Savanna Wet’ climate with an average annual temperature is 28.6 °C. A study by Raj et al. (2019) on cities that lie within 20 kms from Amaravati, showed that the annual mean urban heat island (UHI) intensity varied between 3−4 °C. Thereby increasing the cooling demand in the region. Besides, Amaravati is promoted as India’s first greenfield smart city that is envisioned to reduce ambient temperature by up to 3°C and up to 30 % reduction in energy demand through energy efficient building designs (see supplementary material for the proposed city profile). This greenfield strategy through efficient building design and planning is also central to the ICAP’s space cooling reduction strategy for the next 20 years (see Section 2.3 for details).

Computer-aided design (CAD) model of a chosen residential housing area within the *to-be*-built building stock in Amaravati smart city was generated (see Fig. 3). It was done to replicate a possible scenario of the affordable residential neighbourhood under the Pradhan Mantri Awas Yojana (Housing for All 2022) program by the Government of India (Ministry of Housing & Urban Affairs, 2020a). This scenario was chosen to remain coherent with the future residential building stock estimation for space cooling demand projections as per the ICAP (Ministry of Environment Forest & Climate Change, 2019).

**Fig. 4:**
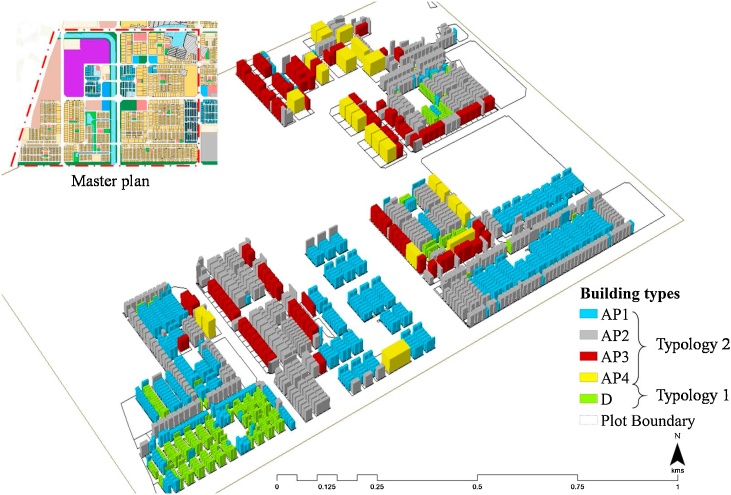
3D model of the study neighbourhood from the city *to-be built* (source: Authors).

Land-use plot maps available from https://gis.apcrda.org/lps/index.html were used to generate the future building stocks in the study area (se Fig. 4). Since most of the affordable housing will be built within the R3 zone, which will allow medium to high-density residential development (see Fig. 3), this zone was chosen for generating future buildings. Local building regulations like the allowable floor space index (FSI), maximum building coverage (%), permissible setbacks and zoning regulations were extracted from the Model Building Bye-Laws 2016 of GoI – Andhra Pradesh Building Rules, 2017 (MAUD, 2017). The regulations were utilised to generate the building footprint polygons. The building footprints covered an area of 310 acres (125.5 ha), containing 2052 residential plots. Out of these 2052 plots, there are two typologies of the buildings. Typology 1 has 425 buildings of detached housing type and remaining 1627 are multi-storied apartment type buildings (Typology 2, see Table 1). Similar typologies were also mentioned in the space cooling demand projections in the ICAP (Ministry of Environment Forest & Climate Change, 2019, pp 20).

**Table 1:** Morphology parameters for various building types.

Minimum-plot size (in m^2^)	Typology	Category of Building as per masterplan	Maximum Building Coverage (%)	Maximum FSI
100−300	Typology 1	Detached house (D)	60	1.75
300−500	Typology 2	Apartment type 1 (AP1)	60	1.75
500−2000		Apartment type 2 (AP2)	60	2
2000−4000		Apartment type 3 (AP3)	50	2
4000−6000		Apartment type 4 (AP4)	50	2.4

The building footprints were generated such that each land plot consisted of only one building with a prescribed built form and associated building characteristics. Since the affordable housing will only be built in land-plots within a prescribed threshold, plots with an area less than 6000 m^2^ and having the number of apartments less than 150 per building were considered. It was assumed that each building utilised the maximum allowable building coverage with minimum permissible setbacks. Building height was calculated as per the maximum FSI assigned to the plot corresponding to the allowable building coverage. The floor height of each apartment was assumed to be 3 m. For simplicity, the permissible building coverage area was assumed as the total built-up area of each floor. Table 1 describes the permissible building regulations associated with each land plot less than 6000 m^2^.

### 3.3 Urban heat island (UHI) simulation

Buildings contribute significantly to increasing the surface temperature that can cause urban heat stress (Santamouris, 2014). The 3-dimensional neighbourhood generated in the Section 3.2 (see Fig. 4) was used to study the formation of urban heat islands (UHI) in the region. UHI formation is significantly related to the multiple neighbourhood morphological and metrological factors like sky view, height of the buildings, aspect ratio of the canyon, canyon radiative geometry, thermal properties of the urban materials, anthropogenic heat release, form factor of the neighbourhood, and temperature difference of urban area from its rural counterparts (Bardhan, Debnath, Malik, & Sarkar, 2018; Mehrotra, Bardhan, & Ramamritham, 2019). To factor in the built environment-induced heating of the generated study area (see Fig. 4), an UHI algorithm was developed in Python v3.7.4 platform to simulate the UHI intensity. UHI intensity is defined as the temperature difference between the urban areas and the rural environment, i.e. the difference between the temperature in a built-up location and the temperature for the same location if no urbanization had taken place. The empirical basis for the algorithm is adopted from (Montavez, Gonzalez-Rouco, & Valero, 2008), as

(2) $$\Delta T_{u-r}=a+b{\text{ѱ}}_{s}+(c+d{\text{ѱ}}_{s})\Delta L↓$$

(3) $${\text{ѱ}}_{s}=\;\text{c}\text{o}\text{s}(a\;\text{t}\text{a}\text{n}(2H/W))$$

where,

${ѱ}_{s}$= sky view factor

$\Delta T_{u-r}$= temperature difference between urban and rural environment (°C)

H/W= height and width of the canyon

H= height of the buildings (m)

W= average width of the streets in front of the building (m)

a, b = coefficients functions of the thermal admittance of the rural ${(\mu }_{r})$ and urban $\left(\mu_{u}\right)$ environments

c, d= coefficients functions of the thermal admittance of the urban $\left(\mu_{u}\right)$environments

ΔL↓= change in downward sky radiation due to anthropogenic and local greenhouse effects (Wm^−2^)

The UHI simulation was prepared based on the following assumption. It was assumed that the *to-be*-built city of Amaravati will have brick walls based on the bioclimatic characteristics of the region (Kotharkar & Bagade, 2018). The thermal inertia of the brick wall was 1139.69 Jm^−2^s^-0.5^ K^-1^ (Balaji, Mani, & Venkatarama Reddy, 2013). This region has a black cotton soil (APCRDA, 2015) which has high thermal inertia as that of a moist soil (Cheruy, Dufresne, Aït Mesbah, Grandpeix, & Wang, 2017). Montavez et al. (2008) recommended using 2200 Jm^−2^s^-0.5^ K^-1^ as the lumped thermal inertia for the empirical validity of their UHI model (see Eq. (2)). Based on these values, the thermal admittance coefficient functions (*a, b, c and d*, see Eq. (2)) were obtained as 1.5, 2.3, 0.08567 and 0.00845, respectively. For simplicity, ΔL↓was assumed to be 30 Wm^−2^ after (Montavez et al., 2008).

The height and width ratio (H/W) of the built form in the hypothesised city (see Fig. 4) was estimated using a GIS extension model called THIS (Tool for Heat Island Simulation) algorithm, after (Nakata-Osaki, Souza, & Rodrigues, 2017) in ArcGIS v10.7.1platform. The THIS model used a variable factor $Z_{0}$ for determining the (H/W) ratio, which is defined as Eq. (4). The input (H/W) ratio was used to calculate the $\Delta T_{u-r}$ as per Eq. (2).

(4) $$Z_{0}=0.5\;H\;(A^{\text{*}}/A^{\text{'}}\;)$$

where,

$Z_{0}$= roughness length (m)

H= average height of buildings in the urban block (m)

A*= is the vertical surface average area facing the canyon (m^2^)

$A^{'}$= average area occupied by each building of the urban block, the horizontal projection (m^2^)

The final air temperature profile of the urban areas for a month was estimated by adding the average night temperature of the nearest rural area (Narukullapadu, approximately 5 km from Amaravati) to the difference in urban-rural temperatures obtained from UHI simulation. The diurnal temperature data was obtained from www.accuweather.com and India Meteorological Department for the year 2019.

### 3.4 Energy demand estimation under UHI constraints

The mean average temperature was expected to rise for the study area due to urban heat island (UHI) effects, which was assumed to increase the night-time temperature. Consequent, the cooling demand was expected to increase rapidly that poses an energy security threat to similar future cities of the Global South (Mastrucci et al., 2019). In this step the estimated nocturnal rise in temperature from the UHI simulations (see Section 3.3) was used to calculate the cooling demand of buildings in the study area.

The key assumptions that were made in this calculation are as follows. The air temperature was assumed to be constant from 6 pm to 6 a.m. for all days in the analysis. The effects of heat gains occurring from night temperature were taken into consideration and no assumptions were made for any trapped head due to solar gains during the day-time.

The cooling demand was estimated using a customised thermal balance equation for buildings (see Eq. (5)). It was based on the thermal-physical building energy model by Fichera et al. (2016). The modified equation to estimate cooling demand for buildings in this study area is as follows,

(5) $$D^{C}=0.024∙C^{DD}\left(G^{ht}-L^{v}\right)+\eta ∙G^{in}$$

where,

$D^{C}$= cooling demand of each building during night (in kWh. m^−2^ year)

$C^{DD}$= cooling degree days

$G^{ht}$= heat gain coefficient (W. K^−1^)

$L^{v}$= heat loss coefficient due to ventilation and infiltration (W. K^−1^)

$G^{in}$= internal heat gains due to occupants and appliances (W. m^−2^)

η= global efficiency of the cooling system

The cooling demand was calculated as the difference between total heat gains and total heat losses. For majority of the year, outdoor temperature during night was higher than desired indoor temperature for hot and humid city like Amaravati. This led to the occurrence of transmission gains, while the heat loss in buildings was accounted for via ventilation and infiltration losses. The heat gain (see Eq. (6)) and heat loss (see Eq. (7)) were calculated for each building using the following formula:

(6) $$G^{ht}=\sum_{i}A_{i}.U_{i}$$

where, $A_{i}$ (m^2^) is the area of the *i*th building envelope component, and $U_{i}$ (W/m^2^K) the thermal transmittance of the *i*th component.

(7) $$L^{v}=\;\rho_{a}.c_{a}.q_{ve}[W/K]$$

where, $\rho_{a}.c_{a}$ is the heat capacity of air per volume $\rho_{a}.c_{a}$ = 1200 J/m^3^K, and $q_{ve}$ is the air flow rate in m^3^/s. We set the average air changes per hour (ACH) to 0.5 h^−1^ with closed windows and 1.5 h^−1^ with open windows. These values corresponded to the air changes rate for natural ventilation with windows on one façade only. It was assumed to be opened during the day and the night with an open area factor of 0.1 and 0.5, respectively, according to ISO 13792 (ISO13792:2012, 2012, ISO13792:2012, 2012).

(8) $$G^{in}=\;\sum_{i}Ig_{i}.A_{i}$$

where, $I_{g_{i}}$ denotes internal gain of the *i*th building (W/m^2^) and $A_{i}\;(m^{2})$ is the inhabited area of the *i*th building (see Eq. (7)). The value for internal gain was taken as the sum of internal gains due to *i*th dweller ($I_{p_{i}})$ and appliances ($I_{a_{i}})$ in the *i*th building for a particular month (see Eq. (9))

(9) $$I_{g_{i}}=\;\sum_{i}\left(I_{a_{i}}+I_{p_{i}}\right)$$

The expression for $I_{a_{i}}$ (see Eq. (10)) was derived from the average energy consumption data for a similar multi-family apartment in the same hot and humid region in India, after (Garg, Shukla, Maheshwari, & Upadhyay, 2014). Based on it, the average energy consumption for a year was reported to be 43 kW h/m^2^/year (or 0.00491 kW h/m^2^/hr), out of which 33 % of the energy consumption was estimated to be used for space cooling purposes (Garg et al., 2014; Kanagaraj et al., 2014). Morever, the residential load curves of Garg et al. (2014) showed that 52 % of the space cooling requirement was during the night. It was also assumed that out of total floor area (i.e. the inhabited area in a house), 80 % of the area was served by the space cooling equipment (Garg et al., 2014). Therefore, the modified empirical estimate of $I_{a_{i}}$ for the *i*th building is defined as (see Eq. (10))

(10) $$I_{a_{i}}=\;\sum \left\{(0.52\text{*}0.00491\text{*}0.33)\text{*}(0.8\text{*}A_{i})\text{*}N_{i}\text{*}\left(12\text{*}n_{d}\right)\right\}$$

where, $A_{i}\;(m^{2})$ is the inhabited area of the *i*th building, $N_{i}$ is the number of *i*th house in the neighbourhood and $n_{d}$ is the number of days in the month

Similarly, for the estimation of $I_{p_{i}}$, the average household size for the residential neighbourhood was assumed to be 3.7 with a carpet area of 100 m^2^ (APCRDA, 2017, pp 20–21). And the internal gain per dweller was assumed to be a static value of 120 W (for light work in a seated position) with a heat gain factor of 1 per dweller (Engineering ToolBox, 2004). Therefore, $I_{p_{i}}$ can be expressed as (see Eq. (11)),

(11) $$I_{p_{i}}=\;\sum \left(120\text{*}3.7\text{*}n_{i}\right)$$

where, $n_{i}$ is the number of households in *i*th building. As per Eq. (4), the expression for $C^{DD}$ for incorporating the urban heat island (UHI) effect into the night-time cooling demand need is illustrated as Eq. (12),

(12) $$C^{DD}=\left(T_{out}-T_{in}\right)\text{*}12\text{*}number\;of\;months$$

where, $T_{out}$ is the night-time outdoor temperature of the study area, and $T_{in}$ is the night-time indoor temperature of the residential units under study. The $C^{DD}$ was found to be positive for March to November, and further energy analysis was performed for this period. The building modelling parameters used in the energy analysis is illustrated in Table 2.

**Table 2:** Lumped building construction parameters for UHI-induced energy analysis.

Position	Parameter	Value
Roof	U value	Same as external wall value
Room	Indoor Temperature	23 °C
	Habitable surface	80 %
	Ventilation	2/hr
	Thermal capacity air	0.34 Wh/m^3^K
	Average store height	3 m
External wall	U value	1.7 W/m^2^ K
Window	Glazing ratio of facade	30 %
	U value	5.23
	Frame	15 % of window area
Thermal bridges	External loss	area*0.05 W/m^2^ K
Cellar	Losses Reduction factor	0.60
	Real Usage Factor	0.95

The daily energy demand ($D^{Total})$ of each building depended on the distribution of cooling load and gains arising due to non-space cooling loads across 24 h. Its day and night breakup was estimated using the load curves (Garg et al., 2014) and total energy demand for a month was calculated as per the Eq. (13),

(13) $$D^{Total}=\left(\frac{D^{C}}{0.52}\right)+\;\left(\frac{G^{in}}{0.74}\right)$$

### 3.5 Estimation of rooftop solar potential

A generalised assumption was made that all the buildings in the study area were composed of flat roof houses with rooftop area equal to that of the building area. The fractions of roof area available for solar PV were assumed to be 50 % of the overall rooftop area. Similar assumptions were made by Singh and Banerjee (2015) for rooftop solar potential estimation in Mumbai, India from satellite-based building footprint calculation. Additionally, to include the shading and other generation losses, a reduction factor of 0.3 was applied (after (Pillai & Banerjee, 2007)). Therefore, the net rooftop area (m^2^) available for the installation of the solar panel ($A_{PV})$ is illustrated in Eq. (14),

(14) $$A_{PV}=0.5\text{*}0.3\text{*}Building\;area$$

The module efficiency (e) of solar cells was assumed to be in coherence with Indian operating conditions of c-Si photovoltaics measured under STC conditions (AM1.5 spectrum, 1000 W/m^2^, 25 °C) as 13 % with system losses of 7.5 % (Apostolou, Reinders, & Verwaal, 2016; Bick et al., 2018; CERC, 2011, pp 34]. The total monthly energy output (E) of solar PV was calculated using the Eq. (15),

(15) $$E=I_{rd}\text{*}number\;of\;days\;in\;a\;month\text{*}e\text{*}A_{PV}$$

The global horizontal irradiance **(**$I_{rd}$) data of Amaravati, Telangana was obtained from Solargis (2019) with an annual average of ∼5.27 kW h/m^2^/day.

### 3.6 Decentralized energy network optimization

A peer-to-peer solar PV-based decentralised energy network (P2P-DEN) topology was adopted to mitigate energy stress in buildings in the study area and reduce its dependency on the electricity grid. Each building was treated as an energy consumer and a peer producer for its neighbouring buildings. Energy consumption of each building was simulated on a monthly scale to analyse energy transactions between buildings in the study area (see Section 3.4). It was assumed that energy transaction occurs after each building meets their cooling demand requirements and mitigate its energy stress.

To compute the energy interactions between the peers a network theoretic approach was adopted, where, the consumers (buildings) were peer nodes (P), with each node i, for i=1, …, P,had an energy consumption of $C_{i}$ and energy generation of $G_{i}$. The links represented the energy transfers among the peers where a node becomes a peer-based on a neighbourhood criterion defined by a distance d threshold function. Here, peer nodes were linked if they were within a radius distance of 50 m.

The mathematical structure of the P2P-DEN model was described through an adjacency matrix $\left(P+1\right)\;\times (P+1)$ with each element $p_{ij}$, (i,j=1, …, P+1 with i≠j). There was no self-interaction between the nodes. To compute the energy transfer through each node i, an energy surplus function ${ES}_{i}$ was computed, where ${ES}_{i}={G_{i}-C}_{i}$. The ${ES}_{i}$ values could be both positive and negative. While a negative ${ES}_{i}$ meant that the building needed energy from its peer to mitigate the energy demand. Whereas, a positive ${ES}_{i}$ meant that the buildings could become a donor to other buildings. A zero value of ${ES}_{i}$ indicated that there were no need for any energy exchanges. It implied that energy transfer could only happen from a node with positive $ES_{i}$ to a node with negative $ES_{i}.$ Thus, $p_{ij}$ could have the following values,

$p_{ij}=1$; if the energy transfer happened between node i to node j

$p_{ij}=-1$; if the energy transfer happened between node j to node i

$p_{ij}=0$; if no energy transfer occurred.

Additionally, each of the nodes were also connected to the main electricity grid which only provided the remaining energy that the P2P-DEN could not met. Finally, an optimisation was performed of the energy transfer from energy surplus buildings to the non-energy surplus buildings while minimising energy intake from the central grid. Based on it, the objective function of the linear optimisation model of the energy flow from the central grid, $Y_{ij}$ is,

(16) $$min=\sum_{p1,j>0}p_{1}Y_{1j}$$

The function (16) was subjected to two constraints (i) energy surplus was distributed among the peers (see Eq. (17)) and, (ii) all energy flows were non-negative (see Eq. (18)).

(17) $${ES}_{ij}=\sum_{j=1}^{P+1}p_{ij}Y_{ij},\;\forall_{i}=1,\ldots ,\;\left(P+1\right),\;i\neq j$$

(18) $$Y_{ij}>0$$

### 3.7 Deriving sustainability rules for mitigating energy stress: towards distributive energy justice

In order to define the sustainability rules in the residential buildings in the study area, it was critical to identify energy stressed (ESt) building from non-energy stressed (NESt) buildings. As mentioned in Section 1, we define energy stress as ‘the shortage of electricity to meet urban-residential cooling needs under the warming effects of climate change’, per the REST framework (see Fig. 2).

A machine-learning-based decision tree algorithm was used to identify the parameters of “Energy stressed (ESt)” and “Non-energy stressed (NESt)” using the rpart package in R (Therneau, Atkinson, & Ripley, 2019). Here, a binary decision tree was constructed by recursively partitioning the data. Where each partition was represented as a binary decision tree and contained a prediction model within each partition. The decision trees were partitioned using the following rules in the form of IF < antecedent > THEN < consequent > where < antecedent > was a conjunction of conditions based in the values of a variable, and the < consequent > was either “Energy stressed (ESt)” or “Non-energy stressed (NESt)”. This way, the antecedent of a rule referred to the parameter of ESt buildings. A building was considered ESt if its cooling demand was above the median value. Two rules were applied in making the decision:

i If cooling demand > median value, then “Energy stressed (ESt)”
ii If cooling demand < median value and Solar energy potential <= median value, then “Energy stressed (ESt)”

**Fig. 5:**
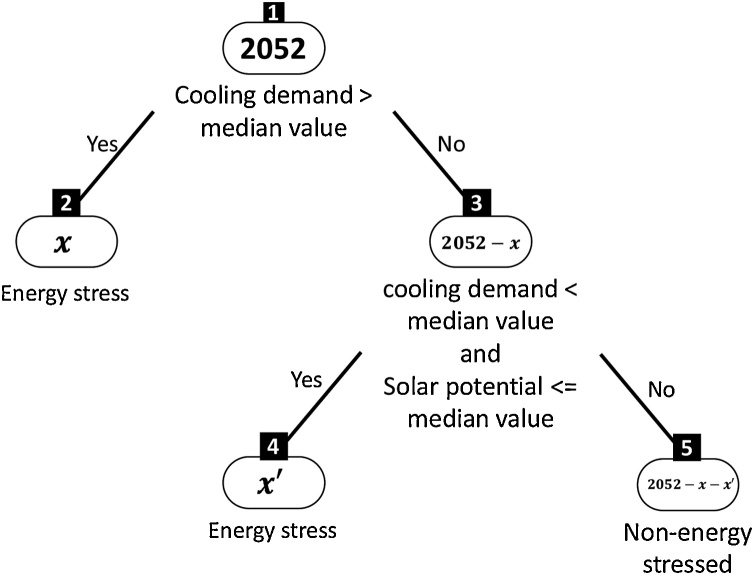
Decision tree to characterise energy stressed and non-energy stressed buildings.

The decision tree contained four leaf nodes, as illustrated in Fig. 5, which contained the point of decision. The decision was which among the 2052 buildings were ESt or NESt. A look-up table characterisation of the NESt buildings was performed to derive the sustainability rules. These sustainability rules were interpreted as way forward for distributive energy justice planning in the study area (see Section 5 for more details).

## 4 Results

This study proposed a Residential Energy Stress (REST) mitigation framework to enable distributive energy justice in cooling demand management in low and middle-income residential buildings of warming cities of the Global South. A proof-of-concept of the REST framework was presented through an empirical investigation of energy stress in a *to-be*-built smart city in India, called Amaravati. The empirical analysis had its basis as per the roadmap for space cooling in residential buildings as per the India Cooling Action Plan (ICAP) (see Ministry of Environment Forest & Climate Change, 2019, pp 19). This section presents the results of cooling energy stress reduction in the Amaravati smart city during the summer months, in order of the seven stages of the REST methodology (Sections 3.1–3.7).

**Fig. 6:**
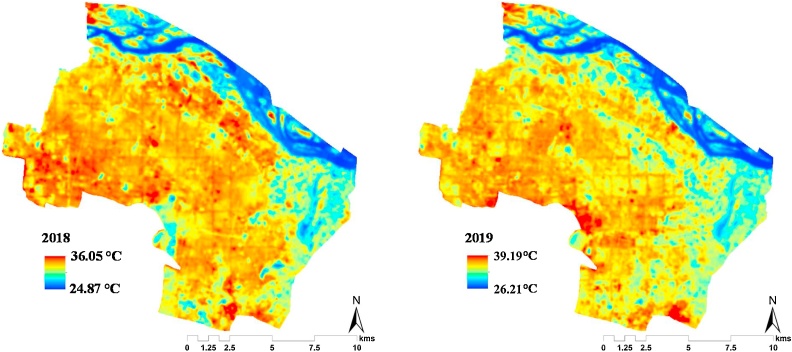
Land Surface Temperature Map for Amaravati, March 2018 and 2019.

The UHI simulations were calculated for all summer months (March to November) in Amaravati, where the cooling demand is expected to peak. The land LST maps (see Fig. 6) generated for Amaravati showed that the temperature ranged between 24.87 °C and 36.05 °C in March 2018 which increased in 2019 where the temperature was within the range of 26.21 °C and 39.19 °C. Temporally, the mean surface temperature was observed to increase by about 2–3 °C, between 2018 and 2019. The average temperature of 2019 was higher than 2018 as construction of the city had already started by 2019 (see Fig. 6). This is in agreement with the previous study on the LST of Amaravati, after Bick et al. (2018b).

**Fig. 7:**
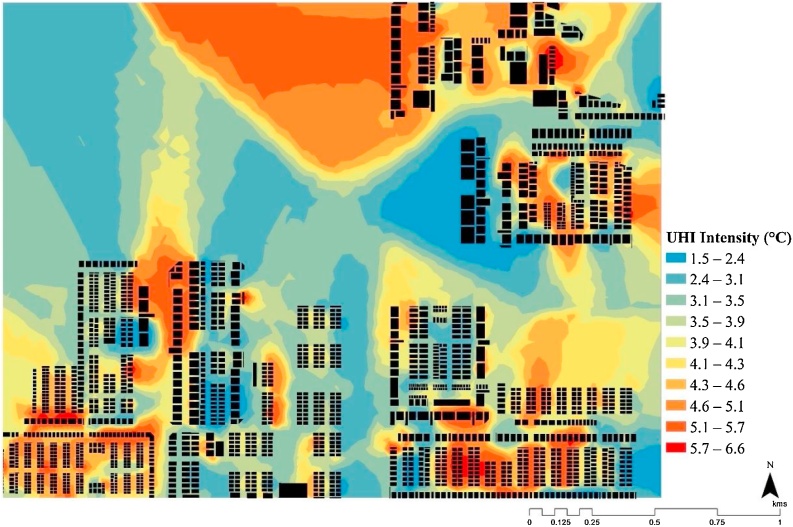
Estimated UHI intensity for March 2019 using Landsat 8 land surface temperature (LST) data.

Most regions within the city (except for the north with dense vegetation and forest) had surface temperature closer to the peak values, were extensively used for agricultural practices. The estimated LST values were found to be comparable to the results of Sundara Kumar, Udaya Bhaskar, and Padma Kumari (2017). The authors found the surface temperature of Amaravati to be within 30 °C and 40 °C, thereby validating our estimates (see Fig. 6). Besides, Kumar, Bhaskar, and Kumari (2016) had also reported that the neighbouring cities of Guntur and Vijayawada had recorded high surface temperatures of 50 °C, indicating that Amaravati might also reach similar levels after the city development had taken place. The hypothetical residential neighbourhood simulated in this study, as per the *to-be*-built city’s masterplan (see Fig. 3), represented the possible urbanisation scenario (see Section 3.2). And, its consequential urban heat island (UHI) effect can be seen in Fig. 7.

The GIS-aided simulation of the night-time outdoor temperature showed a high variability within the range of 1.52 °C and 6.62 °C (see Fig. 7). The impact of temperature around residential buildings may have a possible effect based on the location, plot size, FSI and density of the region (as specified in Table 1). In the residential zone R3 (see Fig. 4), the higher population density sites were expected to experience a higher increase in temperature. A similar UHI induced temperature difference in this region was reported by (Amirtham, 2016) for Chennai (2 °C–4.1 °C) and by Thomas, Sherin, Ansar, and Zachariah (2014) for Kochi (0.62 °C–4.18 °C). It indicated that the *to-be*-built smart city of Amaravati can experience UHI as per our estimation on urbanisation pattern (see Fig. 7).

**Fig. 8:**
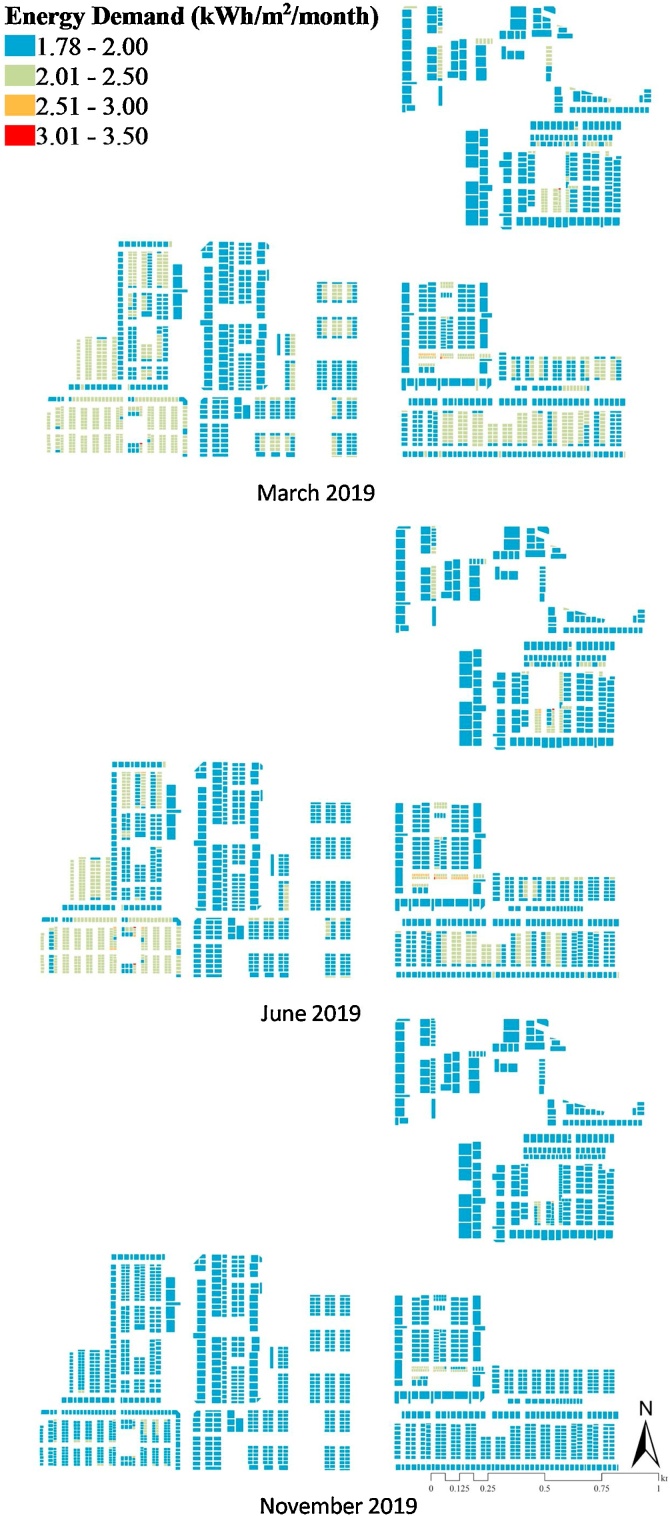
Energy demand prediction for March, June and November 2019 based on the UHI effects.

The predicted energy demand for the neighbourhood is illustrated in Fig. 8 for three months (March, June and November 2019). According to the REST framework (see Fig. 2), this energy demand prediction was performed with consideration of the urban heat island (UHI) effect due to urbanisation in the *to-be*-built smart city of Amaravati. The maximum energy demand estimated for March, June and November is 3.27, 3.48, and 2.77 kW h/m^2^/month, respectively. The typology of the residential buildings (T1 and T2, see Fig. 4) were a control variable to test its impact on the energy and cooling demand. T1 referred to the detached residential houses (n = 425), T2 referred to apartment type buildings (n = 1627); cumulatively there were 2052 buildings. Fig. 8 illustrates the estimated energy demand in these buildings concerning the night-time outdoor temperature as a function of UHI effects.

**Fig. 9:**
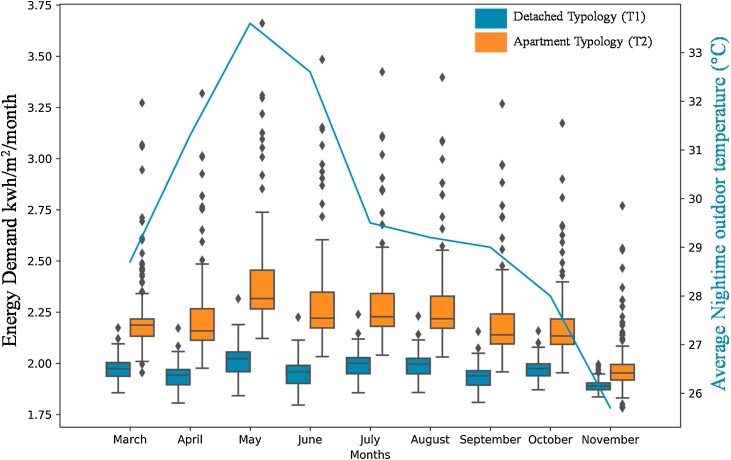
Estimated total energy demand (kWh/m^2^/month) with respect to night-time outdoor temperature (◦C). [Note: T1: Detached buildings (n = 425); T2: Apartment buildings (n = 1627)].

It can be inferred from Fig. 9 that extreme summer temperature influenced higher energy consumption. With the increase in outdoor temperature from March to May (22.7–27.8 °C), the energy demand for both the building typologies increased by 8% and 3% for T1 and T2, respectively, resulting in energy stress. The REST framework, thus, aids in the identification of such urbanisation-factored energy stress. It has specific significance to the India Cooling Action Plan (ICAP, see Ministry of Environment Forest & Climate Change, 2019), as the REST can aid in parametric identification of critical policy variables in the urban-energy nexus (further discussed in Section 5).

**Fig. 10:**
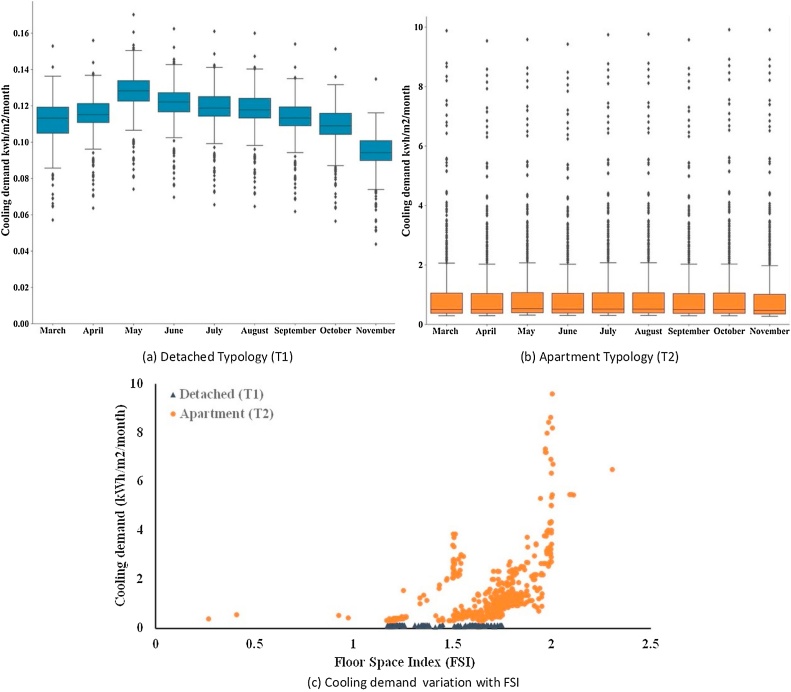
Estimates of cooling demand for T1 (Detached, n = 425) and T2 (Apartment, n = 1627) housing typologies and its variation with the Floor Space Index (FSI).

Fig. 10a and b further illustrate the cooling demand estimation of the two housing typologies. The average cooling demand was highest for May in the detached building type (T1); ∼0.13 kW h/m^2^/month. Similarly, the highest cooling demand in the apartment type buildings (T2) was estimated to be around 0.90 kW h/m^2^/month in May. The cooling demand follows a similar curve as the total energy demand (see Fig. 9, Fig. 10), therefore, indicating a significant impact of UHI on the energy stress in typology. It strengthens the applicability of the REST framework in energy stress reduction estimation in rapidly urbanising scenarios. It also has a crucial implication for the future cities where most of the building stocks are yet to be built, as it can aid in deriving appropriate building/planning guidelines for neighbourhood-scale energy management.

Fig. 10c further visualises the relationship between Floor Space Index (FSI), a critical urban planning variable with the cooling demand for May 2019. The cooling energy consumption in the detached houses (T1, n = 425) is approximately constant, whereas in case of apartment buildings (T2, n = 1627) the cooling demand varies exponentially with the FSI (see Fig. 10c). It illustrates that the FSI can be modulated as a critical urban planning measure to modulate residential cooling energy needs under heat stress (as stated above). This finding adds a crucial planning-based policy variable to energy stress management in warming cities of Global South where most of the building stock is yet to be built. It is also coherent with the recommendations of Mehrotra, Bardhan, and Ramamritham (2019).

**Fig. 11:**
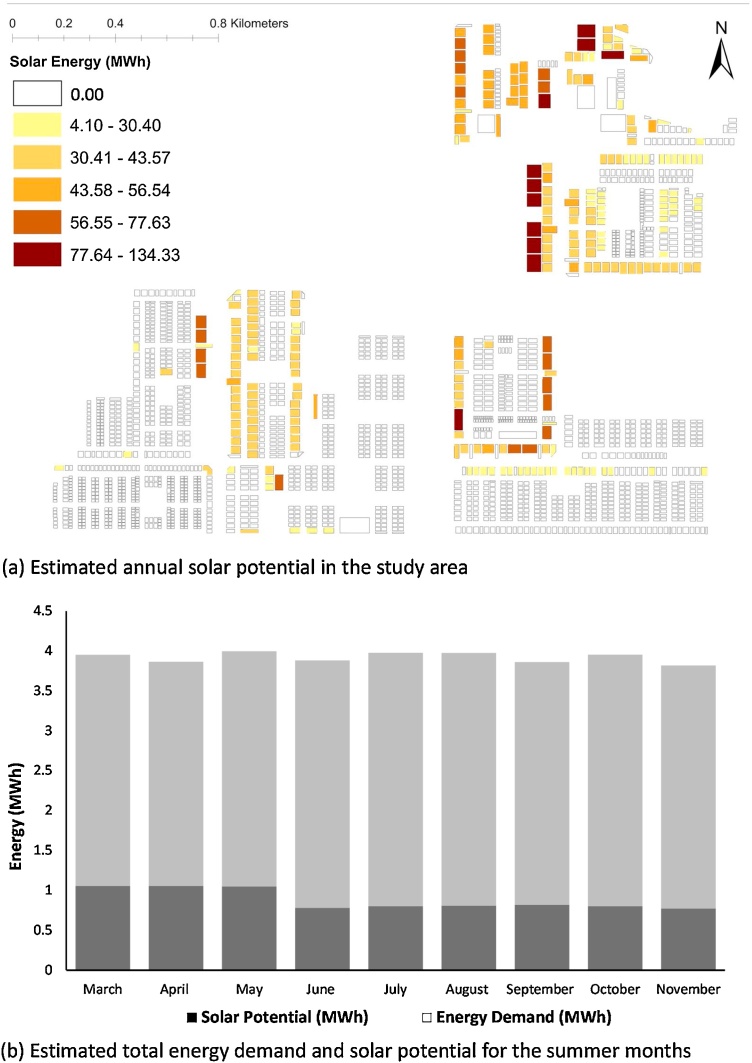
Estimated solar potential in the study area as an energy-stress mitigation strategy.

With the estimation of the cooling demand for the summer months due to UHI effects, the next step in the REST framework (see Fig. 2 and section 3.5) was the estimation of rooftop solar potential in the study area. Addition of rooftop solar PV in cooling energy stress mitigation was a distributive energy justice measure (see Section 2.2. for the rationale). The renewable solution is expected to reduce cooling energy stress due UHI effects without causing significant economic burden in the middle and low-income households under study. Thus, Fig. 11a illustrates the total annual solar potential in the study area. Our estimations show that the rooftop solar PV generation can replace up to 25 % of the total energy demand in the residential buildings for most of the summer months (see Fig. 11b).

**Fig. 12:**
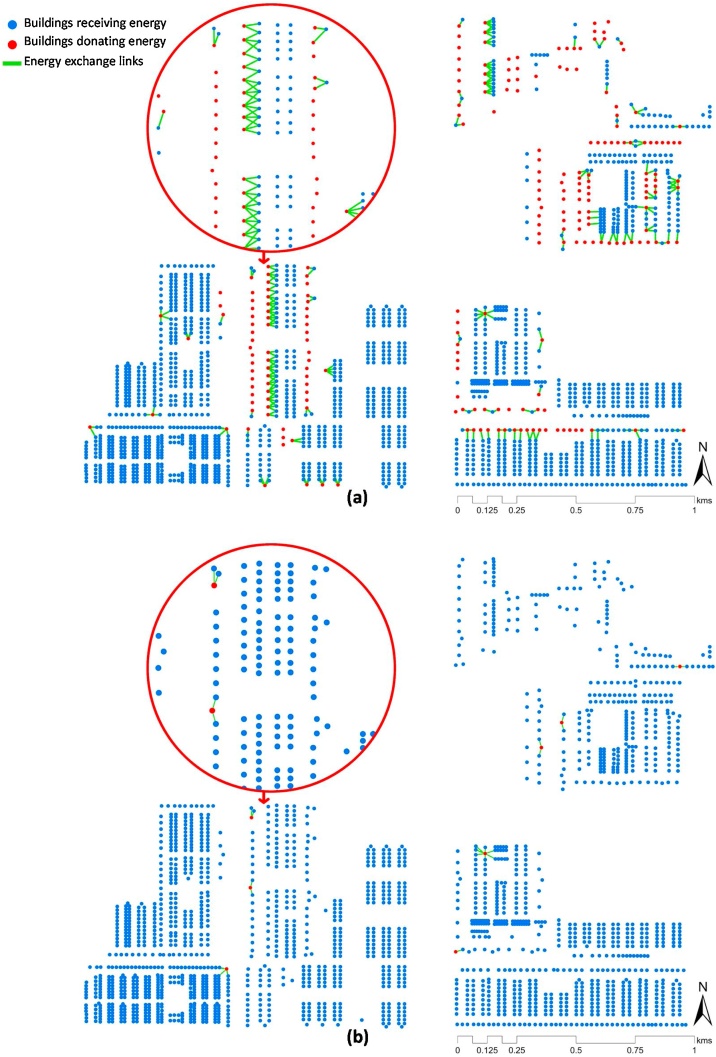
Estimated peer-to-peer energy sharing network for the month of (a) May and (b) November.

Fig. 11 showed that up to 25 % of the energy demand could be met using rooftop solar PV in the study area. It is further supported by the mandate of the government to install mandatory rooftop PV sets in buildings higher than 1000 m^2^ of floor area, as a part of the smart city program (Ministry of Housing and Urban Affairs, 2020a)() and ICAP (Ministry of Environment Forest & Climate Change, 2019). To facilitate this energy substitution with solar PV, the REST framework proposed an optimised P2P decentralised energy network (DEN) (see Sections 3.6 and 3.7). The estimated energy sharing routes based on the optimised-DEN for May and November is illustrated in Fig. 12a and b, respectively.

The optimisation results show that for May, 179 buildings had energy surplus which was shared within the optimised DEN. It resulted in 275 buildings (approximately 13 %) fulfilling its energy demand through P2P DEN. Thus, making them grid independent. The rest of the buildings were then classified as energy stressed (ESt) and non-energy stressed (NESt) based on the decision tree algorithm to derive the sustainability rules (see Fig. 5, Section 3.7). We derive these rules for May as it was the hottest month of the season (see Fig. 8). These sustainability rules represent the outcome of the REST framework that can aid in sustainable urban planning and distributive energy justice policies of the neighbourhood to enable equitable accessibility and affordability of energy for meeting cooling needs. It is even more crucial for rapidly urbanising areas, which are also rapidly warming areas in the Global South.

The result show that 1777 buildings were left after the optimisation process that were dependent on the grid. Out of it, 1628 were classified as NESt buildings that had specific characteristics. The primary characteristics of the non-stressed (NESt) buildings are illustrated in Table 3 that shows minimum plot size should be 100–300 m^2^ if the house is a detached house. The maximum FSI allowed for such houses should be around 1.75. Similarly, for residential apartment types, the most optimised plot size should be between 300–2000 m^2^, and the FSI should be in the range of 1.75–2 (see Table 3).

**Table 3:** Derived sustainability rules of the NESt buildings.

Number of buildings	Minimum-plot size (in m^2^)	Typology	Category of Building as per masterplan	Maximum Building Coverage (%)	Maximum FSI
409	100−300	Typology 1	Detached house (D)	60	1.75
827	300−500	Typology 2	Apartment type 1 (AP1)	60	1.75
392	500−2000		Apartment type 2 (AP2)	60	2

## 5 Discussion

The REST framework is an energy management framework for residential space cooling at a neighbourhood scale. The salient features of this framework were a GIS-based methodology for calculating urban heat island (UHI) derived energy demand in urban areas. And deriving a working methodology for distributive energy justice for mitigating cooling energy stress in residential households, using rooftop solar PV. These two features make this framework critical and unique to the contemporary search on the tools/framework for good cooling energy policymaking. It is even more relevant in current context where most of the building stocks are yet to be built in the rapidly urbanising Global South to meet the housing deficit. The rapid urbanisation will also induce urban heat island affects which will increase the cooling energy needs in middle- and low-income households.

We simulated such a futuristic situation for a *to-be built* smart city in India, called Amaravati. The bioclimatic zone of city was in a hot and humid region of India, that by itself demanded higher energy for cooling. Moreover, factoring in the effects of UHI, our results showed that night-time land surface temperatures can vary between 1.52 °C and 6.62 °C (see Fig. 7). This high variability of LST was due to the urban morphology of the residential houses and the density of the apartments. It illustrated two key points related to energy policy, one being the importance of urban planning variables like floor space index (FSI) and the built-up area as critical energy policy variables. The second point is the inclusion of such variables in the future energy policies, especially policies designed for meeting the increase in cooling energy demand. It is even more important for the Indian case as 80 % of the residential building stock is yet to be built through smart city and affordable housing programs (Ministry of Housing & Urban Affairs, 2020a). These programs are specifically designed for low and middle-income group population, where energy accessibility and affordability are key to improve eudemonic well-being; a premise for distributive energy justice (Samarakoon, 2019). Thus, optimising the urban planning – cooling energy nexus is crucial to enable distributive energy justice in the future cities of the country. Similar inference was also drawn in (Mastrucci et al., 2019; Ministry of Environment Forest & Climate Change, 2019).

The energy estimations were performed for the residential area with two typical building typologies, detached house (T1) and apartment type (T2). Results show high variability in the energy demand in these two typologies, with apartment type being more energy intensive (see Fig. 9). It reveals an important aspect of current urban planning strategy of high-rise development, that intends to maximise occupancy and fulfil the housing deficit (Debnath, Simoes et al., 2020). Future energy policies, especially in India, should consider the cost and benefit of such high-rise development over energy provisioning to the middle-and low-income population groups at an affordable rate. As high-rise development may be crucial for affordable housing objectives, but our energy estimations have showed that cooling demand may increase in vertical building typologies (see Fig. 10).

As an energy stress mitigation strategy, we have used rooftop solar PV in the residential buildings to supply the excess energy needed for cooling due to UHI effects. This strategy is well-documented in the policy roadmaps of Smart Cities planning (Ministry of Housing and Urban Affairs, 2020a)() and India Cooling Action Plan (Ministry of Environment Forest & Climate Change, 2019). The REST framework provided a working plan for the optimised application of rooftop solar PV as a distributive energy justice measure in residential buildings under effect from warming climate. Our estimation showed that up to 25 % of the energy demand could be met using rooftop solar PV in the study area (see Fig. 11). The distributive justice objective was to mitigate energy stress in maximum buildings so that most of the households becomes non-energy stressed (NESt). To achieve this, we have employed a decision tree algorithm (see Section 3.7 for details) and found that 13 % (n = 2052) of the buildings could meet their extra cooling demand through the rooftop solar PV alone, even during the hottest month of May (see Fig. 12). Whereas, the remaining 1777 buildings were still grid dependent even with roof top solar PV (see Section 4).

As a part of the distributive justice model, i.e., making maximum building NESt, we proposed a peer-to-peer sharing (P2P) network topography (see Fig. 5). The network optimisation results (see Fig. 12) showed that out of 1777, 1628 could be made non-energy stressed while connected to both grid and rooftop solar PV system. It was achieved through the neighbourhood level P2P energy sharing. We believe that it has crucial implication on the search for good cooling policy making and adding the perspective of distributive energy justice at the demand side can aid in fulfilling future cooling needs under heat stress in the Global South. Thus, the REST framework, for the first time, materialised a policy roadmap (Indian Cooling Action Plan) for a future smart city and visualised the impact of urbanisation dynamics on cooling energy stress due to UHI.

## 6 Conclusion and policy implication

This study proposed a residential energy stress mitigation framework called REST to support policy decision on energy infrastructure and urban planning under the influence of urbanisation-induced warming effects, especially for the future cities of the Global South. A proof-of-concept of the REST framework was derived through a case study of a *to-be*-built smart city in the hot and humid region of India. A residential neighbourhood was generated based on the master plan of the *to-be*-built city with applied constraints of urban heat island (UHI) formation. It was found that the REST framework could mitigate the energy stress of 1628 out of 2052 buildings, i.e., almost 80 % of the buildings. The results further aided in the derivation of planning-based sustainability rules that can aid in energy stress mitigation. The rules include constraints on the Floor Space Index (FSI) and density of the residential neighbourhood. The sustainable planning bound FSI was found to be 1.75 for detached houses and both 1.75 and 2 for apartment building types. It has critical significance with the India Cooling Action Plan (ICAP, Ministry of Environment Forest & Climate Change, 2019) which specifically demanded future-proofing upcoming building stocks with latest energy efficient building codes and sustainable planning measures. Thus, the REST framework provided the technical foundation for realising objectives of national cooling action plans (like ICAP) with an integrated urban-energy nexus optimisation approach.

Similarly, the most optimised plot sizes for energy stress mitigation were in the range of 100–2000 m^2^, indicating medium housing density pattern. Since housing typology influences energy demand, typology guidelines based on policy variables like FSI, and built-up coverage should be incorporated for realising non-energy stress homes. The spatial distribution of energy demand indicates the favourability for certain land plots over others, indicating that a variable plot price based on its energy generation potential can be introduced. It can lead to capitalisation of energy efficiency in the housing market while providing a greater range of choice for the buyers (Aydin, Brounen, & Kok, 2015). It has a broader policy implications toward realising distributive energy justice at the ground level by enabling energy efficiency and cooling equity in the low and middle-income housing market. It can ensure that the future building stocks and neighbourhood planning has an embedded element of energy stress mitigation capacity, such that everyone can access and afford energy for space cooling under climate and urbanisation-induced heat stress.

The findings of this study and the REST framework has critical implications in the current search of ‘good’ cooling energy policy under the warming effects of climate change and urbanisation in the Global South. The cooling action plan for the next 20 years in India, ICAP, stressed on the need for energy efficient design, strict adherence to building codes and bye-laws and use of renewable decentralised system to meet future cooling demand (Ministry of Environment Forest & Climate Change, 2019). While ICAP was a policy roadmap, the REST framework provides a methodology to derive proof-of-concept for such policy roadmaps. Thus, filling a critical methodology gap of rapidly estimating and mitigating cooling energy stress due to urbanisation effects at a neighbourhood and city scale.

The REST framework enables policymakers and planners to utilise critical urban planning instruments like FSI and urban density in building resiliency towards climate-change-induced weather shocks, like heat stress, and urbanisation-induced local heating like urban heat island (UHI). The energy management solutions derived through the REST framework depends entirely on just use of renewable resources; the case study presented here was based on an effective decentralised network of rooftop solar PV that could mitigate energy stress of almost 80 % of the households. It further strengthens the policy application of distributive energy justice perspectives in contemporary energy management problems (like managing future cooling demand). In doing so, researchers have used urban greening, rooftop solar PV and green roof as energy management measures under UHI conditions (Poruschi & Ambrey, 2018; Sanchez & Reames, 2019).

The mitigation scheme presented through the REST framework provides an applied energy pathway for enabling distributive energy justice in warming Global South context and fills a significant literature gap. It has critical policy implications in deriving sustainability rules for addressing cooling demand needs in rapidly urbanising and future cities, while at the same time filling the housing deficit through energy efficient and inclusive housing stocks. The future cities of Global South will attract millions of low and middle-income citizens in this decade, and energy security of the nations will depend on the derived pathways that can promote energy justice and better quality of life.

While this study provides a robust proof-of-concept of the REST framework on future urbanisation and cooling energy stress, it remains mostly theoretical at this stage. The limitation of this study lies in the assumptions made in the calculation of energy demand using a static cooling degree day methodology and various other lumped models concerning UHI estimation and city modelling. Future work is needed in deriving a dynamic model of cooling demand calculation at a neighbourhood and city-scale. Further work is also needed in validating the REST model on existing rapidly urbanising cities and calibrating it for improving the reliability of the results. Our current efforts in urban building energy modelling (UBEM) are developing newer methods in addressing it and validating the model using data-driven approaches. In doing so, we aim to integrate REST in a digital twin model, so that urban energy processes and flows can be accurately integrated in the model. Better robustness of the REST framework can also be established through energy investigation of a rapidly urbanising and over-crowded city like Mumbai, Rio de Janeiro, Abuja, Nairobi etc. to name a few, in the Global South that is going to be the economic powerhouse in this decade. It can enable in the discovery of critical policy variables that can address the global challenge of sustainable cooling.
