# Supplementary material for: REST framework: A modelling approach towards cooling energy stress mitigation plans for future cities in warming Global South
Source: Sustain Cities Soc. 2020 Oct;61:102315. doi: 10.1016/j.scs.2020.102315 (PMC7493751; doi:10.1016/j.scs.2020.102315)
Supplement: Supplementary file 1 [file mmc1.docx]

**Supplementary material**

City Profile: Amaravati, the *to-be built* smart city in India

Amaravati is a to-be-built city, and future state capital of Andhra Pradesh is aspiring to become a leading smart city of India with a total area of 217.23 km2. Located at 16.54° N 80.52° E, the city is surrounded by Krishna River in its North and Tadepalli hills in the West. The location provides the strategic benefit of water resources and biodiversity that can integrate into the city’s fabric and creating its regional green network. The presence of green and blue belt will help in offsetting increase in local temperatures due to urbanisation. It is three phase construction due by 2050 and is projected to accommodate a population of 4.5 million while generating a 1.8 million employment by 2050. The quality of life in the city is envisioned to be high and to meet international standards. There are elaborate plans for enhancing public transport driven mobility, energy efficiency and sustainability through renewable energy systems, waterfront accessibility, water monitoring and sustainability systems and Net Zero Waste production (APRCDA, 2017). The land use and land cover (LULC) map of this to-be-built city are illustrated in Fig 3.

Environmental sustainability and resource management plans for the *to-be built* city

The World Bank-supported ‘Amaravati Sustainable Capital City Development Project’ (ASCCDP) has three main objectives at its core. These include the provision of adequate infrastructure for urban and rural dwellings, climate-resilient development for urban spaces and technical assistance for high-quality living. These initiatives, along with the nationwide concern towards sustainability, have been reinforced through national and state-level policy interventions including National Environment Policy-2006, Andhra Pradesh State Action Plan for Climate Change 2012, Energy Conservation Building Code of India (ECBC). At the city level, World Bank Environmental and Social Safeguard Policies [1] are adopted to mitigate the adverse effect of the project on indigenous communities and croplands, preserving natural habitats and physical resources of the region. [1] are adopted to mitigate the adverse effect of the project on indigenous communities and croplands, preserving natural habitats and physical resources of the region. However, the developments are accompanied by massive scale conversion of land to non- agricultural use, deforestation and pollution generated through infrastructure operation. Despite the existing institutional structure and policies, the city faces risk towards inadequate resources and techniques for the proper execution of its plans. It can result in capacity breakdown to provide secure environment and quality lifestyle to citizens (APRCDA, 2017).

The state of Andhra Pradesh is one of the worst affected states due to heat waves from years 2005 to 2015 claiming many lives [2], [3]. There has also been an increasing trend (with 95% significance) in annual mean maximum and minimum temperature between 1951-2010 in this region [4]. Studies have shown that the land surface temperature at Vijaywada and Guntur, the nearest cities to Amaravati, has been as high as 50 °C during daytime in 2016 [5]. Amaravati is likely to experience such events due to change in land use, rise in anthropological activities and climate change. It can have predisposed health burdens on the citizens and energy security loopholes. As mentioned in section 1, this study tries to investigate existing heat stresses at Amaravati and possible future stress due to microclimatic changes and propose the REST framework as an evidence-based policy instrument. The state of Andhra Pradesh is one of the worst affected states due to heat waves from years 2005 to 2015 claiming many lives [2], [3]. There has also been an increasing trend (with 95% significance) in annual mean maximum and minimum temperature between 1951-2010 in this region [4]. Studies have shown that the land surface temperature at Vijaywada and Guntur, the nearest cities to Amaravati, has been as high as 50 °C during daytime in 2016 [5]. Amaravati is likely to experience such events due to change in land use, rise in anthropological activities and climate change. It can have predisposed health burdens on the citizens and energy security loopholes. As mentioned in section 1, this study tries to investigate existing heat stresses at Amaravati and possible future stress due to microclimatic changes and propose the REST framework as an evidence-based policy instrument.

Envisioning to be a leading smart city, Amaravati has adopted ‘Efficient Resource Management’ as one of the six major goals towards sustainability (APRCDA, 2017). Deriving from the principles of smart grid, decentralisation, renewable energy and green buildings, the city plan incorporates regulations for future construction for affordable housing types. The bye-laws [6] have suggested use of solar power for water heating for residential plots with sizes larger than 300 m2. [6] have suggested use of solar power for water heating for residential plots with sizes larger than 300 m2. Solar energy-driven lighting of common areas, energy-efficient HVAC systems and installation of photovoltaic cells (PV) is approved for plots larger than 1000 m2. This research tries to investigate the impact of such regulations on residential buildings in Amaravati and derive sustainability rules.

References

[1] TheWorldBank, “Environmental and Social Policy,” Environmental and Social Policies, 2018. .

[2] National Institute of Urban Affairs, “Heat waves,” 2016.

[3] G. J. Van Oldenborgh et al., “Extreme heat in India and anthropogenic climate change,” Nat. Hazards Earth Syst. Sci., vol. 18, no. 1, pp. 365–381, 2018.

[4] L. S. Rathore, S. D. Attri, and A. K. Jaswal, “State level climate change trends in India,” Indian Meteorol. Dep., vol. Meteorolog, no. No. ESSO/IMD/EMRC/02/2013, pp. 1–147, 2013.

[5] K. Sundara Kumar, P. Udaya Bhaskar, and K. Padma Kumari, “Assessment and Mapping of Urban Heat Island using Field Data in the New Capital Region of Andhra Pradesh, India,” Indian J. Sci. Technol., vol. 10, no. 11, pp. 1–8, 2017.

[6] MAUD, “Model Building Bye Laws 2016 of GoI – Andhra Pradesh Building Rules,” 2017.
